# Protective Effects of Small-Molecule Oligopeptides Isolated from Tilapia Fish Scale on Ethanol-Induced Gastroduodenal Injury in Rats

**DOI:** 10.3390/nu13062078

**Published:** 2021-06-17

**Authors:** Jiani Hu, Rui Liu, Xiaochen Yu, Zhen Li, Xinran Liu, Yuntao Hao, Na Zhu, Jiawei Kang, Yong Li

**Affiliations:** Department of Nutrition and Food Hygiene, School of Public Health, Peking University, Beijing 100191, China; hujiani95@163.com (J.H.); liuruipku@163.com (R.L.); xiaochenyu46@126.com (X.Y.); lizhenbjmu@163.com (Z.L.); liuhappy07@163.com (X.L.); haoyuntaolly@163.com (Y.H.); summer920503@163.com (N.Z.); kjiawei@yeah.net (J.K.)

**Keywords:** tilapia collagen oligopeptides, gastroduodenal injury, antioxidant, anti-inflammation, anti-apoptosis

## Abstract

Peptic ulcer has a serious impact on people’s health around the world, and traditional medicines can cause adverse reactions. This study investigated the protective effects of tilapia collagen oligopeptides (TCOPs) on gastroduodenal injury. Seventy-two specific pathogen-free (SPF) male Sprague Dawley (SD) rats were randomly divided into six groups according to body weight: normal control group, ethanol group, whey protein group (500 mg/kg BW), and three TCOPs dose groups (250, 500, 1000 mg/kg BW). After intragastric administration for 30 days, the acute gastroduodenal injury was induced by anhydrous ethanol (5 mL/kg, intragastrically) in all groups except the normal control group. Biomarkers in gastric and duodenal tissue and serum were measured. Furthermore, western blot was used to detect the expression of apoptosis-related proteins. The results showed that the administration with TCOPs significantly reduced gastric and duodenal ulcer index, increased gastric juice pH, superoxide dismutase (SOD), catalase (CAT), and glutathione peroxidase (GSH-Px) activities, along with the reduction of malondialdehyde (MDA) contents. TCOPs decreased tumor Necrosis Factor-α (TNF-α), interleukin-1β (IL-1β), and myeloperoxidase (MPO) levels, while interleukin– 10 (IL-10) levels were increased. Furthermore, pepsinogens 1 (PG1), pepsinogens 2 (PG2), gastrin (GAS), and the pepsinogen ratio (PGR) were decreased, the prostaglandin E2 (PGE2) and NO contents were increased after TCOPs intervention. Moreover, TCOPs up-regulated the expression of Bcl-2 and inhibited the expression of Bax and Caspase-3. In conclusion, TCOPs have protective effects on ethanol-induced gastroduodenal injury through gastrointestinal mucosal microcirculation promotion, antioxidation, anti-inflammation, and anti-apoptosis mechanisms.

## 1. Introduction

With the rapid development of society, people’s lifestyles and eating habits have gradually changed. Gastrointestinal diseases in the population have been widespread. Peptic ulcer is now one of the most serious gastrointestinal diseases, which not only seriously affects the health and quality of life of contemporary people, but also leads to a large health care cost and social burden and is considered a global public health problem [1]. Peptic ulcer disease is a multifactorial and complex disease involving gastric and duodenal ulcers, mainly occurring in the stomach and duodenal bulb. It is generally accepted that peptic ulcer is the result of an imbalance between the protective factors of the gastric and duodenal mucosa and the factors that cause gastrointestinal mucosal damage [2,3,4].

Invasive factors including hydrochloric acid, pepsin, smoking and drinking, helicobacter pylori infection, non-steroidal anti-inflammatory drugs, certain dietary habits, and psychological stress can cause gastrointestinal mucosal lesions, which in severe cases can lead to ulcers [5,6]. Alcohol consumption worldwide increases as people’s material well-being increases, and the number of alcohol-related gastrointestinal diseases increases year by year [7]. The damage of ethanol to the gastric and duodenal mucosa is involved in many aspects and complex mechanisms. High concentrations of ethanol into the digestive tract can increase vascular permeability, edema formation, and epithelial cell loss, leading to significant gastrointestinal mucosal damage [8,9]. It has been demonstrated that ethanol can damage the gastrointestinal mucosa by promoting gastric acid secretion, inducing oxidative stress, activating inflammatory pathways, and affecting normal mucosal microcirculation and cell apoptosis [10,11]. Clinical use of synthetic drugs such as proton-pump inhibitor and receptor antagonist in the treatment of gastroduodenal diseases, but long-term use can cause side effects such as poor healing of the stomach and recurrence of ulcers and adverse effects [12,13,14].

There is growing evidence that bioactive peptides have a wide range of physiological activities and play an essential role in improving human health [15]. Natural bioactive peptides with multiple biological mechanisms play a vital role in preventing and treating gastric and peptic ulcer diseases. For example, a collagen peptide derived from the skin of Cod (Gadus macrocephalus) has a significant protective effect against acetic acid-induced gastric ulcers [16]. Fish provides a rich source of collagen with high nutritional quality and is an ideal source for the preparation of collagen peptides [17,18]. Many studies have shown that bioactive peptides derived from Tilapia have various unique physiological functions, such as antioxidation, anti-aging, lowering blood pressure, immune regulation, antibacterial activity, and the ability to repair tissue defects [19,20,21,22]. Furthermore, tilapia collagen oligopeptides (TCOPs) extracted from tilapia have lower molecular weight, better digestibility and absorption [19,23], and great value in the prevention and treatment of gastroduodenal injury. Whey protein is a recognized high-quality protein, which contains all 20 amino acids that make up proteins. It is mainly composed of β-lactoglobulin, α-whey protein, and immunoglobulin, as well as other protein components. It has multifarious health benefits, such as boosting immunity, inhibiting cancer cell formation, and reducing intestinal strain [24]. The whey protein group was used as a positive control to eliminate the false-positive results caused by extra protein intake.

This study investigated the possible protective effects of TCOPs on ethanol-induced gastroduodenal mucosa injury in rats and explored its potential mechanism by biomolecular assay.

## 2. Materials and Methods

### 2.1. Preparation of TCOPs

Tilapia collagen oligopeptides: solid white powder, provided by Shengmeinuo Biotechnology Co., Ltd. (Hainan, China), mainly composed of oligopeptides with molecular weight less than 1000; the specific molecular weight distribution was <180 Da (9.97%), 180~500 Da (56.06%), 500~1000 Da (26.67%), which was a mixture of small molecular active peptides obtained from tilapia scales by enzymolysis technology. The detailed amino acid composition of the sample is shown in Table 1.

### 2.2. Animals

Seventy-two healthy SPF male SD rats aged from 6 to 8 weeks. Provided by the Peking University Health Science Center. Reared in the SPF animal house, with a temperature range of 22 ± 2 °C, relative humidity of 50%~60%, and time between day and night of 12 h: 12 h. The animals ate freely during the experiment. Laboratory animal production license number: SCXK (Beijing) 2016-0010, Laboratory animal use license number: SYXK (Beijing) 2016-0041. The animal feeding management and the experimental operation followed the experimental animal ethics and the regulations of the Beijing Municipality on the administration of experimental animals.

### 2.3. Ethanol-Induced Gastric and Duodenal Mucosa Injury Model

Rats were randomly divided into six groups according to body weight: normal control group, ethanol group, whey protein control group (250 mg/kg BW), and collagen oligopeptide low, medium, and high dose group (250, 500, 1000 mg/kg BW, respectively referred to as TCOPs-L, TCOPs-M, TCOPs-H). The subjects were given daily by gavage, the normal control group and ethanol group were given distilled water, the whey protein group and collagen peptide group were given corresponding concentrations of the subjects. After the 30th day of administration, all experimental animals were strictly forbidden to eat (without water) for 24 h, during which the subjects were also forbidden to be given. The next day, after 1 h of intragastric administration of each dose group, except the normal control group, the other groups were given anhydrous ethanol (5 mL/kg) by intragastric gavage and anesthetized with pentobarbital 1 h later, and the rats were euthanized. Subsequently, gastroduodenal injury assessment and related biochemical indexes were determined.

### 2.4. Macroscopic Observation and Evaluation of Gastric and Duodenal Mucosa Injury

The whole stomach was cut along the greater curvature of the stomach, the duodenum was cut along the mesentery, and the contents were rinsed with cold physiological saline. The gastric and duodenal tissues were flattened with the surface facing upwards to evaluate the injury of the gastroduodenal mucosa. The gastric ulcer index was calculated by Guth’s improved method [25,26]. As shown in Table 2.

On the mucosal side of the duodenum, the degree of peptic ulcer was evaluated as follows [27]: 0: no lesion; 1: ulcer area with the longest diameter <2 mm; 2: the longest diameter is 2~5 mm; 3: the longest diameter is 6~8 mm; 4: the longest diameter >9 mm, 5: ulcer perforation.

### 2.5. Histopathological Analysis

After macroscopic observation, the gastric and duodenal tissues were immersed in 10% formalin solution for 24 h, then dehydrated in 95% ethanol. The tissues were embedded in paraffin wax, cut into 5 μm thickness, and stained with hematoxylin and eosin (H&E). The pathological observation and evaluation of the whole layer of gastrointestinal mucosa were performed by fluorescence microscope (E400; Nikon, Tokyo, Japan).

### 2.6. Measurement of Gastric Juice 

Gastric juice and contents were collected in a test tube. The supernatant was then centrifuged at a speed of 3000 rpm for 10 min. The volume of the supernatant was measured accurately with a 10 mL cylinder, and the pH value of gastric juice was measured with a micro-pH meter.

### 2.7. Enzyme-Linked Immunoabsorbent Assay

Fresh gastroduodenal tissue was taken, and the inflammatory factors (IL-1β, TNF-α, IL-10, MPO) levels and oxidative stress biomarkers (SOD, GSH-Px, CAT, MDA), and the PGE2 and NO contents in gastric and duodenal tissues in different dose groups were determined by ELISA according to the instructions of the kit (Nanjing Jian Cheng Bioengineering Institute, Nanjing, China). Blood was taken from the femoral artery of rats, the blood sample was kept at 4 °C, and then the supernatant was extracted by centrifugation at the speed of 3500 r/min for 10 min. The PG1, PG2 levels, and serum gastrin in rats were detected according to the instructions of the test kit. 

### 2.8. Western Blot 

Two hundred microliters of protein lysates was added to every 10 mg tissue and ground to homogenate for complete cleavage. The protein was extracted at 4 °C and centrifuged for 10 min at 12,000 rpm. The concentration of protein was determined according to the operating instructions of the BCA protein quantitative reagent kit (Sigma, St. Louis, MO, USA). The 20 ug sample was subjected to electrophoresis on a sodium lauryl sulfate-polyacrylamide gel. After electrophoresis, the separated proteins were transferred to a polyvinylidene fluoride membrane (Millipore, Billerica, MA, USA). The membrane was immersed in 5% skimmed milk powder prepared with TBST and sealed for 4 h, followed by incubation with primary antibodies against Bcl-2 (ab194583; Abcam, Cambridge, UK) at 1:1000 (loading control), Bax (ab32503; Abcam, Cambridge, UK) at 1:5000, Caspase-3 (#9662; CST, Danvers, MA, USA) at 1:1000, and β-actin at 1:2000 (ab8227; Abcam, Cambridge, UK) overnight at 4 °C, Then, a blocking solution was used to dilute HRP-labeled secondary antibodies, the membranes were incubated with secondary antibody goat anti-rabbit IgG (1:10,000) at room temperature for 4 h, and the HRP was incubated with the membrane for 4 h after the membrane was diluted with the sealing solution. Visual detection using ECL chemiluminescence developer (Millipore, Billerica, MA, USA). Finally, Image-Pro Plus (IPP) software (Media Cybernetics, Rockville, MD, USA) was used for quantification and processing.

### 2.9. Statistical Analysis

The data were expressed as ± standard deviation ($\bar{x}\pm s$). Using IBM SPSS 24.0 software (IBM, Armonk, NY, USA) for one-way analysis of variance, the variance homogeneity test was conducted for those with significant differences. The variance homogeneity test was conducted for those with the LSD method to analyze the differences between the average values of each experimental group and the control group. If the variance was not up to the standard, we used the rank-sum test to do the statistical analysis. The difference was significant with *p* < 0.05.

## 3. Results

### 3.1. Effect of TCOPs on the Appearance and Ulcer Index of Gastric and Duodenal Mucosa in Rats 

**Figure 1:**
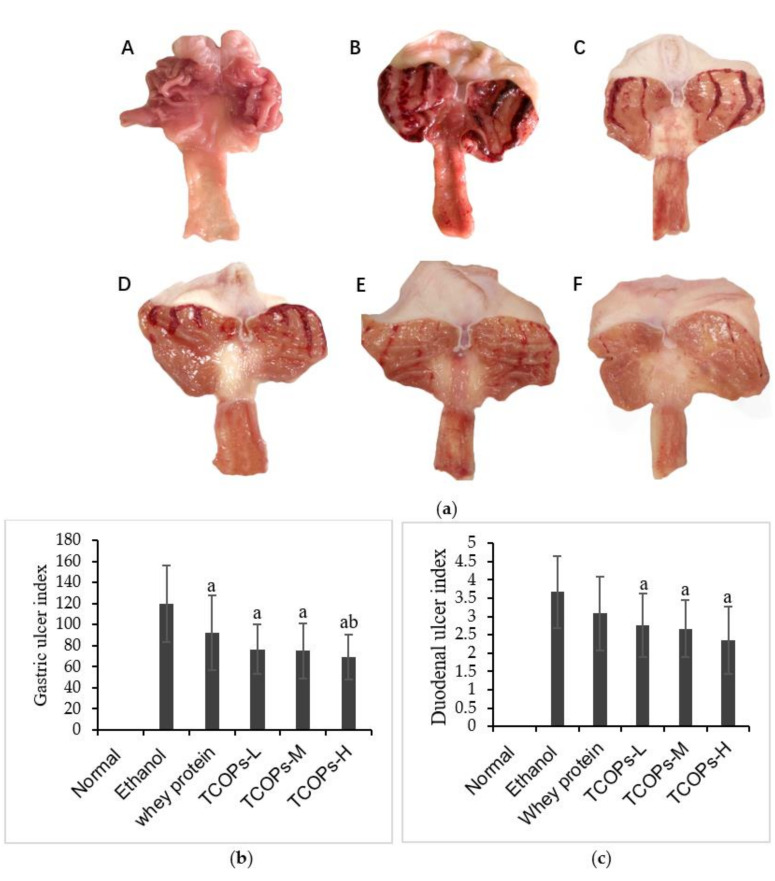
Effect of tilapia collagen oligopeptides (TCOPs) on gross evaluation (**a**) and ulcer index of gastric (**b**) and duodenal (**c**) mucosa in rats. Values are expressed as mean ± SD (*n* = 12). a: means compared with the ethanol group, *p* < 0.05; b: means compared with the whey protein group, *p* < 0.05. **A**: normal control; **B**: ethanol; **C**: whey protein; **D**: TCOPs-L, 250 mg/kg of tilapia collagen oligopeptides group; **E**: TCOPs-M, 500 mg/kg of tilapia collagen oligopeptides group; **F**: TCOPs-H, 1000 mg/kg of tilapia collagen oligopeptides group.

As shown in Figure 1a, the surface of gastric mucosa in the normal control group was pink, with no hyperemia or edema, and the gastric mucosa was smooth and complete. After the model was established by anhydrous alcohol, the gastric mucosa of rats in each group was damaged in different degrees, and the injury rate was 100%. In the ethanol group, the gastric mucosa was the most severely injured, with apparent bleeding injury, dark red surface of the mucosa, and lacunar or lamellar bleeding. Compared with the ethanol group, the gastric mucosal injury was significantly reduced in the TCOPs dose group, and the length and width of the bleeding band were decreased. The gastric mucosa injury of rats treated with the high dose of TCOPs was the least and significantly better than those in rats pretreated with whey protein. The naked eye observation of rats’ duodenal tissue showed that, compared with the normal control group, bleeding and ulcer damage were observed in the other dose groups. Compared with the ethanol group, hemorrhagic damage of the duodenum of rats pretreated with TCOPs was reduced to different degrees.

The gastric mucosal injury index is shown in Figure 1b. Compared with the ethanol group, the integral injury index of the TCOPs dose group was significantly decreased (*p* < 0.05), and the ulcer index of the TCOPs-H group was significantly lower than those of the whey protein group (*p* < 0.05). The inhibition rate of gastric injury was 41.96%. The duodenal ulcer index is shown in Figure 1c. The duodenal ulcer index of the ethanol group was significantly higher than those in the normal control group, and TCOPs significantly decreased the ethanol-induced duodenal ulcer index of rats (*p* < 0.05), the inhibition rate of the TCOPs-H was 35.69%, and the inhibition effect was the best.

### 3.2. Effect of TCOPs on Gastric and Duodenal Histomorphology in Rats

**Figure 2:**
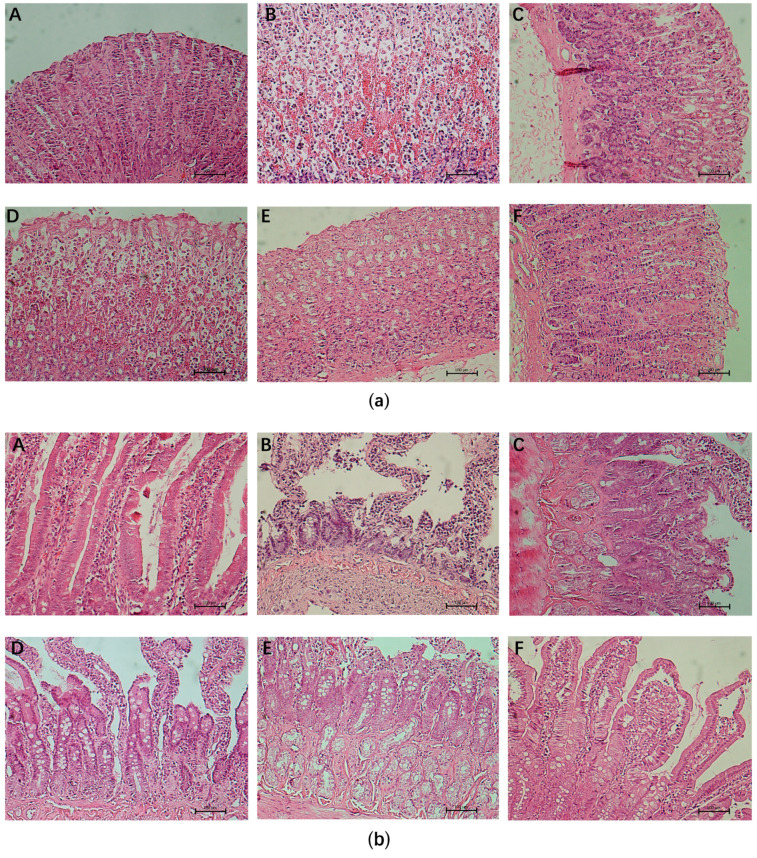
Effect of tilapia collagen oligopeptides (TCOPs) on gastric (**a**) and duodenal (**b**) histomorphology in rats. Photomicrographs of HE (magnification 200 ×) stained sections from gastroduodenal mucosa. **A**: normal control; **B**: ethanol control; **C**: whey protein, 250 mg/kg of whey protein; **D**: 250 mg/kg of TCOPs-L; **E**: 500 mg/kg of TCOPs-M; **F**: 1000 mg/kg of TCOPs-H.

As shown in Figure 2a, in the normal control group, the structure of each layer of the gastric mucosa was complete and clear, the gastric mucosa was smooth, the cell morphology and the structure of the gland were complete and regular. The gastric mucosal layer of rats in the ethanol control group was extensively injured, which was characterized by massive cell necrosis, obvious cell congestion and edema, incomplete gland structure, and inflammatory cell infiltration. The gastric mucosal morphology of rats pretreated with whey protein and TCOPs was improved to varying degrees, and congestion and edema were reduced. 

As shown in Figure 2b, In the normal control group, the structure of the duodenal mucosa was complete and smooth, the intestinal villi were dense and arranged regularly, the central lacteal duct was clearly visible, and the cells and intestinal glands were neatly arranged. In the ethanol control group, incomplete epithelial structure, severe destruction of intestinal villi, the disappearance of central lacteal duct, lysis of most small intestinal glands and denatured, and necrosis and peeling-off of epithelial cells were observed. The injury degree of the whey protein group was slightly reduced, and the intervention of TCOPs significantly improved the histopathological damage caused by ethanol.

### 3.3. Effect of TCOPs on Gastric Juice in Rats 

As shown in Table 3, anhydrous ethanol significantly decreased the pH of gastric juice and promoted the secretion of gastric juice compared with the normal control group. Compared with the ethanol group and whey protein group, the pH of gastric juice in TCOPs-H was significantly increased (*p* < 0.05), while the volume of gastric juice was significantly decreased (*p* < 0.05) in TCOPs-M and TCOPs-H.

### 3.4. Effects of TCOPs on Antioxidant Activity and Lipid Peroxidation Index in Gastric and Duodenal Tissue of Rats

As shown in Table 4 and Table 5, compared with the normal control group, the SOD, CAT, and GSH-Px activities in gastroduodenal tissues of the ethanol group were significantly decreased, while the MDA contents were markedly increased (*p* < 0.05). Nevertheless, compared with the ethanol group, the SOD, CAT, and GSH-Px activities in gastroduodenal tissue of TCOPs-M and TCOPs-H were significantly improved, and the MDA contents were significantly decreased (*p* < 0.05). Moreover, in comparison to the whey protein group, pretreatment with TCOPs showed SOD activities in gastroduodenal tissue and CAT activities in gastric tissue significantly increased, whereas TCOPs significantly reduced the MDA contents in gastroduodenal tissue (*p* < 0.05).

### 3.5. Effect of TCOPs on Inflammatory Factors in Gastroduodenal Tissue of Rats

As shown in Table 6 and Table 7, after administration with anhydrous ethanol, the TNF-α, IL-1β, and MPO levels in gastroduodenal tissue were significantly elevated while IL-10 levels were significantly decreased (*p* < 0.05). Additionally, in comparison to the ethanol group, the TNF-α, IL-1β, and MPO levels in gastroduodenal tissue of TCOPs groups were significantly reduced (*p* < 0.05). Conversely, the IL-10 levels in gastroduodenal tissues of TCOPs groups were markedly increased (*p* < 0.05); pretreatment with TCOPs considerably decreased the TNF-α and IL-1β levels in gastroduodenal tissue as well as the MPO levels in duodenal tissue, while the IL-10 levels in gastric tissues were significantly increased compared to the whey protein group (*p* < 0.05).

### 3.6. Effect of TCOPs on Mucosal Barrier Function in Rats 

As shown in Table 8, the serum PG1, PG2, and GAS contents of the ethanol group were significantly higher than those of the normal control group (*p* < 0.05), and the PG1, PG2, and GAS contents of TCOPs groups were significantly lower than those of the ethanol group, while PGR increased significantly (*p* < 0.05); compared with whey protein group, the serum PG1 contents in TCOPs-H as well as GAS contents in TCOPs-M and TCOPs-H decreased significantly (*p* < 0.05).

### 3.7. Effect of TCOPs on PGE2 and NO in Gastroduodenal Tissue of Rats

As shown in Table 9, the PGE2 and NO contents in gastroduodenal tissue of the ethanol group were significantly lower than those of the normal group (*p* < 0.05), the PGE2 and NO contents in gastroduodenal tissue of TCOPs groups were elevated remarkably compared to the ethanol group (*p* < 0.05); compared with whey protein group, the administration of TCOPs showed a significant increase in NO and PGE2 contents of gastric and duodenal tissue. (*p* < 0.05). 

### 3.8. Effect of TCOPs on the Expression of Apoptosis-Related Proteins in Gastroduodenal Tissue of Rats

**Figure 3:**
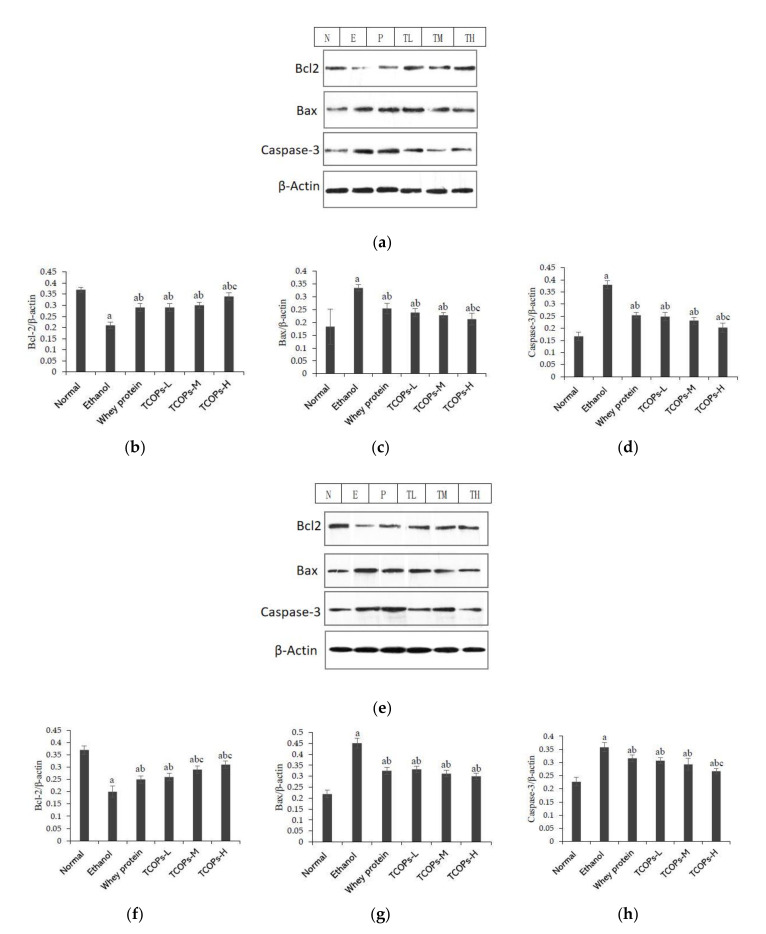
Effect of TCOPs on the expression of apoptosis-related proteins in gastrointestinal tissue of rats. (**a**) Effect of TCOPs on the expression of apoptosis-related proteins in gastric tissue of rats; (**b**) the expression of Bcl-2 in gastric tissue of rats; (**c**) the expression of Bax in gastric tissue of rats; (**d**) the expression of Caspase-3 in gastric tissue of rats; (**e**) effect of TCOPs on the expression of apoptosis-related proteins in duodenal tissue of rats; (**f**) the expression of Bcl-2 in duodenal tissue of rats; (**g**) the expression of Bax in duodenal tissue of rats; (**h**) the expression of Caspase-3 in duodenal tissue of rats. a: means compared with the normal control group, *p* < 0.05; b: means compared with the alcohol group, *p* < 0.05; c: means compared with the whey protein group, *p* < 0.05.

As shown in Figure 3, the expressions of Bcl-2, Bax, and Caspase-3 in gastrointestinal tissue of the ethanol group was significantly lower than those of the normal control group (*p* < 0.05). Furthermore, compared with the ethanol group and the whey protein group, the expression of Bcl-2 in gastroduodenal tissues of rats pretreated with TCOPs increased observably, while the Bax and Caspase-3 levels decreased significantly (*p* < 0.05).

## 4. Discussion

As an example of gastroduodenal mucosal injury, peptic ulcer is a disease causing increased morbidity and mortality among humans worldwide. It is a heterogeneous disease with multiple etiologies, and it has been shown that heavy drinking is associated with gastrointestinal disorders. The primary pharmacological treatment of the disease is anti-gastric acid secretion drugs, but long-term use of these drugs treatment can have a variety of side effects [12,28]. Finding new therapies that produce minimal side effects or no side effects is the focus of medical research to prevent gastrointestinal injury effectively. Peptides are structural and functional fragments of proteins. Small molecule oligopeptides are generally composed of less than 10 amino acids. Compared with proteins, small molecular peptides are characterized by low molecular weight, high absorbability, high bioavailability, and low sensitization and exist in proteins as important bioactive components. In addition, amino acids, like a small pearl, are very limited in function and are easily saturated with absorption. If they are linked into a peptide chain, they will have their own special biological activity. Presently, a variety of natural active ingredients are widely used in research to prevent gastrointestinal diseases [29,30,31,32]. Collagen peptides derived from marine fish have great potential in preventing and treating gastroduodenal diseases due to their excellent antioxidant [33,34], cytogenetic, and tissue repair abilities [35].

In this study, we investigated the protective effects of TCOPs on ethanol-induced gastroduodenal injury in rats. Among the destructive factors, ethanol is a significant external factor. Ethanol has both hydrophobic alkyl groups and hydrophilic hydroxyl groups in its molecular structure, which can destroy the barrier defense system of gastric mucosa and weaken the ability of gastric mucosa to defend against the invasion of gastric acid, bile, and many digestive enzymes. It plays a role in the destruction of gastric and duodenal mucosal cells [36]. The animal model of ethanol-induced gastrointestinal injury has been widely used as an experimental model to study the pathophysiological mechanism of gastrointestinal mucosal injury [37,38]. We refer to previous studies combined with pre-experiments. It was found that 5 mL/kg of anhydrous ethanol could cause significant damage to the gastroduodenal mucosa without causing death in rats. Therefore, an acute gastroduodenal injury model was established with this dose [26,39].

In this study, compared with the normal control group, the ethanol group of rats’ gastroduodenal mucosa damage significantly severe, the injury rate was 100%, and injury index significantly increased. It resulted in visible bleeding, edema, and erosion of the gastroduodenal tissue. When rats were pretreated with TCOPs, the gastroduodenal hemorrhage induced by ethanol was significantly reduced, and the pathological changes of gastroduodenal mucosa were improved considerably, especially in the high-dose group. These results demonstrate that TCOPs have beneficial effects on ethanol-induced gastric and duodenal lesions.

The involvement of acute alcohol-induced oxidative stress in the pathogenesis of gastrointestinal mucosal injury has been demonstrated by many studies [40]. Ethanol can induce oxidative stress through various pathways, including the production of reactive oxygen species; oxidative stress plays a vital role in the development of gastric diseases such as gastric adenocarcinoma, peptic ulcer, or gastritis [41]. In this regard, the gastrointestinal mucosa, through a series of endogenous antioxidant defense systems, resists the invasion of harmful substances. Nonenzymatic and enzymatic antioxidants have antioxidant defenses, and ethanol inhibits the activities of these enzymes in the gastrointestinal mucosa, leading to the accumulation of hydrogen peroxide and lipid oxidation, which ultimately leads to the loss of membrane integrity [42]. SOD and CAT are major antioxidant enzymes, and the levels of SOD and CAT are essential indicators of the ability to scavenge free radicals. Furthermore, GSH-Px is one of the crucial antioxidant enzymes. Its primary physiological function is to clear the body of free radicals. Ethanol-induced gastrointestinal injury is often accompanied by a significant decrease in GSH-Px level, mainly due to the oxidation of GSH-Px after the formation of ethanol-induced superoxides or the combination of acetaldehyde and GSH-Px from the oxidation of alcohol [43]. Lipid peroxidation is thought to be a significant factor in the development of alcohol-induced oxidative damage to the gastrointestinal mucosa, and MDA is a considerable indicator of lipid peroxidation. The activities of SOD, CAT, and GSH-Px in the ethanol group were significantly decreased, and the MDA contents were significantly increased, which indicated that oxidative stress and lipid peroxidation reaction occurred in the gastric and duodenal tissues of rats. The activities of SOD, CAT, and GSH-Px in gastroduodenal tissues were significantly increased, and MDA levels were significantly decreased in TCOPs groups. The results showed that pretreatment with TCOPs significantly enhanced the activity of antioxidant enzymes and inhibited the activity of lipid peroxidation. It can effectively relieve acute ethanol-induced oxidative stress in gastroduodenal mucosa.

Inflammation is a momentous pathogenesis of gastrointestinal injury. When the gastrointestinal tract is stimulated by alcohol, the inflammatory cells overreact and produce many inflammatory factors [44,45]. TNF-α and IL-1β are crucial inflammatory cytokines. External stimuli may stimulate the innate immune system, leading to the release of inflammatory cytokines such as TNF-α and IL-1β, enhancing cell apoptosis and neutrophil migration by promoting oxygen free radicals and the caspase cascade pathway and eventually lead to severe gastrointestinal mucosal damage [46]. MPO is a marker of neutrophil infiltration, which indicates the degree of inflammation. This is because the inflammatory tissues are heavily infiltrated by neutrophils, which contain MPO, which is involved in the development of inflammation [47]. IL-10 plays an important role in down-regulating the inflammatory cascade by enhancing anti-inflammatory cytokines and slowing down the production of pro-inflammatory cytokines and preventing autoimmune diseases. The expression of TNF-α, IL-1β, and MPO may be increased after gastrointestinal mucosa injury [48]. In this study, the levels of TNF-α, IL-1β, and MPO were significantly increased, and the levels of Il-10 were decreased considerably in the gastric and duodenal tissues of the ethanol group after ethanol stimulation of the gastroduodenal mucosa leading to injury and inflammation. However, the intervention of TCOPs significantly reduced the levels of inflammatory factors in rat gastroduodenal tissues, ameliorated the infiltration of inflammatory cells, reduced the inflammatory response, and showed a good anti-inflammatory effect to inhibit the injury in gastroduodenal. Furthermore, alcohol may increase the expression of H^+^-K^+^-ATP on the cell membrane. Ethanol can stimulate parietal cells to overproduce gastric acid, thus reducing the pH value of gastric juice and increasing gastric juice volume [49]. The results showed that the gastric juice secretion of rats pretreated by TCOPs decreased significantly. The pH value of gastric juice increased to different degrees, significantly different from that of the ethanol group. This result is consistent with the previous study [50]. These results suggest that TCOPs can reduce gastric acid invasion on gastric and duodenal mucosa by affecting gastric juice secretion and have a good protective effect on the gastric and duodenal tissue.

Pepsinogen is an inactive precursor of pepsin secreted by the principal cells of the gastric mucosa. They are classified as PGI and PGII in biochemistry and immunochemistry and are converted to active pepsin by the action of gastric acid or activated pepsin, PGI, and PGII, and the ratio of PGI to PGII (PGR) levels reflect the functional and morphological status of the gastric mucosa [51]. Pepsinogen is more sensitive to damage to the gastric mucosa, and in general, when PGI and PGII are higher than normal, the risk of gastric mucosa injury is higher [52]. The decrease in PGR is currently used to detect atrophic gastritis [53]. In this study, the ethanol group had significantly higher pepsinogen levels than the normal control group, and the pepsinogen levels were significantly decreased after intervention with TCOPs, suggesting that TCOPs can protect gastroduodenal mucosa by reducing the pepsinogen levels. GAS can stimulate gastric acid secretion. Excessive gastric acid secretion can lead to gastric mucosal permeability changes and accelerate the formation of ulcers. In this study, GAS levels increased significantly in the ethanol group. Still, GAS levels decreased significantly in all dose groups of TCOPs, indicating that TCOPs can protect gastrointestinal mucosa by decreasing GAS levels.

A variety of PGs help maintain mucosal integrity, a phenomenon known as “gastric cell protection”. PGE2 promotes the flow of gastric mucosal microcirculation, stimulates the secretion of hydrogen carbonate, mediates adaptive immune protection, increases protein synthesis and cell regeneration, and enhances the resistance of gastric mucosal cells to strong stimuli [54,55], and ultimately enhances the ability to repair damaged gastrointestinal mucosa [56]. NO and PGE2 are known as vasodilators, which can inhibit platelet aggregation and thrombosis and accelerate the flow of gastrointestinal mucosal microcirculation. NO is thought to act with PGE2 on the regulation of gastric mucosal integrity and acidity and participate in the inhibition of neutrophil aggregation and the increase of blood flow. The results showed that the levels of PGE2 and NO in the ethanol group were significantly lower than those in the normal control group, suggesting that ethanol inhibits the synthesis and secretion of endogenous NO and PGE2 and participates in the damage of the gastroduodenal tissue. However, TCOPs can increase the levels of PGE2 and NO in acute alcohol-induced gastroduodenal injury and play an important role in maintaining gastroduodenal microcirculation and reducing gastroduodenal injury.

Previous studies have shown that ethanol-induced gastrointestinal injury is closely associated with apoptosis, which breaks down the gastrointestinal mucosal barrier and eventually leads to peptic ulcers [57]. Oxidative stress in the ulcer region can initiate the intrinsic pathway of apoptosis and activate pro-apoptotic protein Bax. The translocation of activated Bax to mitochondria stimulated the formation of apoptotic bodies and finally activated the executor of apoptosis, Caspase-3 [58]. Anti-apoptotic members of the Bcl-2 family block the release of Cytochrome C, and Bcl-2 binds to pro-apoptotic proteins or interacts directly with Bax to prevent mitochondrial pore formation [59]. In this study, compared with the normal control group, the expression of Bax and Caspase-3 was significantly increased. The expression of Bcl-2 was decreased in the gastric, duodenal tissues of the ethanol group, suggesting that ethanol-induced gastroduodenal injury was related to the mechanism of apoptosis. Compared with the ethanol group, pretreatment with TCOPs significantly decreased the expression of Bax protein and increased the expression of Bcl-2 protein, indicating that TCOPs reduced ethanol-induced mitochondrial apoptosis. In addition, pretreatment with TCOPs reduced the expression of caspase-3 in rat gastric, duodenal tissues. Among them, the high dose of TCOPs had the best regulatory effect on anti-apoptotic protein Bcl-2 and pro-apoptotic protein Bax and caspase-3. Therefore, this study suggests that the protective effect of tilapia collagen oligopeptide on ethanol-induced gastroduodenal injury may be related to the reduction of apoptosis.

## 5. Conclusions

This study is the first to identify the protective effects of TCOPs on alcohol-induced gastroduodenal injury in animals. The results of this study show that in ethanol-induced acute gastroduodenal injury, TCOPs can significantly reduce the degree of gross gastroduodenal injury. TCOPS can obviously protect mucous membrane barrier and improve microcirculation through antioxidant and anti-inflammatory effects, reducing serum pepsin and gastrin contents, and increasing PGE2 and NO contents in gastroduodenal tissue. Furthermore, TCOPs can modulate the expression of apoptotic factors of the gastroduodenal tissue (Bcl-2, Bax, and Caspase-3), thus reducing apoptosis of the cells in gastroduodenal tissue. The results of this experiment provide a scientific basis for the development of TCOPs in the prevention, treatment, and health care of gastroduodenal mucosal injury and have the potential to be an ideal adjuvant therapy for alleviating gastroduodenal mucosal injury. Still, the corresponding effect also needs to carry on the more thorough discussion and verification in the crowd experiment.

## Figures and Tables

**Table 1:** The Amino Acids Composition of TCOPs.

Amino Acids	Content (g/100 g)	Amino Acids	Content (g/100 g)
Aspartic Acid	5.44	Cystine	0.043
Glutamic Acid	11.22	Valine	2.34
Serine	2.83	Methionine	1.06
Histidine	0.75	Phenylalanine	1.90
Glycine	23.67	Isoleucine	1.30
Threonine	2.77	Leucine	2.62
Arginine	8.34	Lysine	3.40
Alanine	10.24	Proline	10.42
Tyrosine	0.32	hydroxyproline	8.72

**Table 2:** Scoring criteria for gastric ulcer index.

Degree of Erosion	1	2	3	4	
Spot erosion	Each spot	-	-	-	
Erosion length	<1 mm	1–2 mm	2–3 mm	3–4 mm	>4 mm, segmented scored
Erosion width	>2 mm, score doubled				
Total score	Spot Erosion + Linear Erosion Length + (Linear Erosion Width) × 2				

**Table 3:** Effect of TCOPs on gastric juice in rats.

Groups	pH	Volume (mL)
Normal	3.29 ± 0.89	0.96 ± 0.64
Ethanol	1.86 ± 0.46 ^a^	2.81 ± 0.90 ^a^
Whey protein	2.16 ± 0.53 ^a^	2.55 ± 0.91 ^a^
TCOPs-L	2.27 ± 0.67 ^a^	2.27 ± 0.90 ^a^
TCOPs-M	2.42 ± 0.54 ^a^	1.83 ± 0.82 ^abc^
TCOPs-H	2.72 ± 0.42 ^abc^	1.54 ± 0.67 ^bc^

Note: data are shown as mean ± SD, *n* = 12/group. a: means compared with the normal control group, *p* < 0.05; b: means compared with the ethanol group, *p* < 0.05; c: means compared with whey protein group, *p* < 0.05.

**Table 4:** Effects of TCOPs on antioxidant and lipid peroxidation index in gastric tissue of rats.

Groups	CAT (U/mg)	SOD (U/mg)	GSH-Px (U/mg)	MDA (nmol/mg)
Normal	23.07 ± 6.14	96.63 ± 10.98	40.45 ± 8.41	0.14 ± 0.81
Ethanol	14.23 ± 4.38 ^a^	69.28 ± 12.64 ^a^	29.08 ± 9.51 ^a^	0.88 ± 0.11 ^a^
Whey protein	16.75 ± 4.84 ^a^	78.96 ± 15.20 ^a^	31.87 ± 6.85 ^a^	0.78 ± 0.16 ^a^
TCOPs-L	17.11 ± 2.83 ^a^	82.52 ± 14.96 ^a^	33.88 ± 8.20	0.67 ± 0.12 ^ab^
TCOPs-M	19.54 ± 5.09 ^b^	96.55 ± 17.42 ^b^	36.62 ± 7.58 ^b^	0.57 ± 0.14 ^abc^
TCOPs-H	22.24 ± 5.10 ^bc^	91.52 ± 16.50 ^bc^	37.22 ± 8.65 ^b^	0.46 ± 0.16 ^abc^

Note: data are shown as mean ± SD, *n* = 12/group. CAT: catalase; SOD: superoxide dismutase; GSH-Px: Glutathione peroxidase; MDA: malondialdehyde. a: means compared with the normal control group, *p* < 0.05; b: means compared with the ethanol group, *p* < 0.05; c: means compared with whey protein group, *p* < 0.05.

**Table 5:** Effects of TCOPs on antioxidant and lipid peroxidation index in duodenal tissue of rats.

Groups	CAT (U/mg)	SOD (U/mg)	GSH-Px (U/mg)	MDA (nmol/mg)
Normal	24.19 ± 8.01	93.31 ± 10.50	56.13 ± 14.65	1.01 ± 0.56
Ethanol	12.67 ± 7.36 ^a^	67.14 ± 14.97 ^a^	37.99 ± 12.07 ^a^	4.82 ± 0.74 ^a^
Whey protein	16.17 ± 8.06 ^a^	72.51 ± 12.69 ^a^	43.79 ± 14.77	4.55 ± 0.92 ^a^
TCOPs-L	18.22 ± 8.06	78.05 ± 11.92 ^ab^	46.42 ± 17.82	4.06 ± 0.97 ^a^
TCOPs-M	19.89 ± 9.16 ^b^	82.17 ± 8.93 ^abc^	50.86 ± 12.48 ^b^	3.82 ± 1.17 ^abc^
TCOPs-H	21.38 ± 8.77 ^b^	85.45 ± 9.08 ^bc^	51.90 ± 15.99 ^b^	3.03 ± 0.71 ^abc^

Note: data are shown as mean ± SD, *n* = 12/group. CAT: catalase; SOD: superoxide dismutase; GSH-Px: Glutathione peroxidase; MDA: malondialdehyde. a: means compared with the normal control group, *p* < 0.05; b: means compared with the ethanol group, *p* < 0.05; c: means compared with whey protein group, *p* < 0.05.

**Table 6:** Effect of TCOPs on the content of inflammatory factors in gastric tissue of rats.

Groups	TNF-α (pg/mg)	IL-1β (pg/mg)	IL-10 (pg/mg)	MPO (U/g)
Normal	37.48 ± 7.01	60.52 ± 8.60	36.00 ± 7.49	3.20 ± 0.84
Ethanol	104.58 ± 7.38 ^a^	97.75 ± 15.97 ^a^	16.61 ± 8.15 ^a^	6.34 ± 1.28 ^a^
Whey protein	77.11 ± 6.25 ^ab^	86.88 ± 15.23 ^ab^	21.77 ± 8.40 ^a^	4.59 ± 0.79 ^ab^
TCOPs-L	76.85 ± 9.36 ^ab^	82.50 ± 14.92 ^ab^	24.57 ± 8.51 ^ab^	4.57 ± 0.88 ^ab^
TCOPs-M	68.69 ± 8.40 ^abc^	75.08 ± 9.80 ^abc^	28.01 ± 5.17 ^abc^	4.12 ± 0.78 ^ab^
TCOPs-H	66.46 ± 7.72 ^abc^	72.08 ± 8.44 ^abc^	30.45 ± 5.39 ^bc^	4.05 ± 0.86 ^ab^

Data are shown as mean ± SD, *n* = 12/group. TNF-α: tumor necrosis factor-α; IL-1β: interleukin-1β; IL-10: interleukin-10; MPO: myeloperoxidase. a: means compared with the normal control group, *p* < 0.05; b: means compared with the ethanol group, *p* < 0.05; c: means compared with whey protein group, *p* < 0.05.

**Table 7:** Effect of TCOPs on the content of inflammatory factors in duodenal tissue of rats.

Groups	TNF-α (pg/mg)	IL-1β (pg/mg)	IL-10 (pg/mg)	MPO (U/g)
Normal	33.03 ± 6.69	103.62 ± 11.61	80.24 ± 21.89	2.19 ± 0.46
Ethanol	94.48 ± 6.44 ^a^	188.71 ± 19.91 ^a^	29.57 ± 13.72 ^a^	3.47 ± 0.52 ^a^
Whey protein	88.57 ± 6.15 ^a^	167.05 ± 25.03 ^ab^	58.85 ± 19.65 ^ab^	3.22 ± 0.56 ^a^
TCOPs-L	82.86 ± 6.67 ^ab^	159.49 ± 21.00 ^ab^	67.04 ± 24.26 ^b^	3.09 ± 0.19 ^a^
TCOPs-M	75.24 ± 7.33 ^abc^	143.92 ± 13.22 ^abc^	69.93 ± 22.76 ^b^	2.92 ± 0.44 ^ab^
TCOPs-H	69.33 ± 9.55 ^abc^	138.69 ± 23.38 ^abc^	73.55 ± 22.87 ^b^	2.76 ± 0.42 ^abc^

Data are shown as mean ± SD, *n* = 12/group. TNF-α: tumor necrosis factor-α; IL-1β: interleukin-1β; IL-10: interleukin-10; MPO: myeloperoxidase. a: means compared with the normal control group, *p* < 0.05; b: means compared with the ethanol group, *p* < 0.05; c: means compared with whey protein group, *p* < 0.05.

**Table 8:** Effects of TCOPs on the indicators related to mucosal barrier function in rats.

Groups	PG1 (ng/mL)	PG2 (ng/mL)	PGR	GAS (pg/mL)
Normal	17.36 ± 1.81	7.30 ± 0.81	2.41 ± 0.40	38.56 ± 4.23
Ethanol	25.85 ± 2.37 ^a^	12.96 ± 0.88 ^a^	2.00 ± 0.19 ^a^	66.81 ± 4.64 ^a^
Whey protein	23.25 ± 1.57 ^ab^	9.77 ± 1.27 ^ab^	2.42 ± 0.34 ^b^	48.53 ± 5.11 ^ab^
TCOPs-L	23.82 ± 1.65 ^ab^	9.77 ± 0.88 ^ab^	2.45 ± 0.19 ^b^	49.74 ± 5.32 ^ab^
TCOPs-M	23.85 ± 1.31 ^ab^	9.64 ± 0.75 ^ab^	2.49 ± 0.24 ^b^	44.72 ± 3.66 ^abc^
TCOPs-H	21.40 ± 1.17 ^abc^	9.21 ± 0.75 ^ab^	2.34 ± 0.25 ^b^	42.20 ± 3.27 ^abc^

Note: data are shown as mean ± SD, *n* = 12/group. PG1: Propepsin 1; PG2: Propepsin 2; PGR: the pepsinogen ratio; GAS: gastrin. a: means compared with the normal control group, *p* < 0.05; b: means compared with the ethanol group, *p* < 0.05; c: means compared with whey protein group, *p* < 0.05.

**Table 9:** Effect of TCOPs on PGE2 and NO in gastroduodenal tissue of rats.

Groups	Gastric Tissue		Duodenal Tissue	
	PGE2 (pg/mg)	NO (μmol/g)	PGE2 (pg/mg)	NO (μmol/g)
Normal	170.70 ± 10.31	3.21 ± 0.36	248.23 ± 7.45	12.96 ± 0.88
Ethanol	120.18 ± 10.10 ^a^	1.09 ± 0.27 ^a^	167.31 ± 5.97 ^a^	7.30 ± 0.81 ^a^
Whey protein	140.32 ± 7.36 ^ab^	2.01 ± 0.26 ^ab^	200.09 ± 4.41 ^ab^	9.62 ± 0.71 ^ab^
TCOPs-L	139.97 ± 4.73 ^ab^	2.61 ± 0.26 ^ab^	201.96 ± 5.85 ^ab^	10.02 ± 0.76 ^abc^
TCOPs-M	153.26 ± 12.49 ^abc^	2.76 ± 0.34 ^ab^	211.57 ± 5.82 ^abc^	11.07 ± 0.98 ^abc^
TCOPs-H	146.13 ± 8.79 ^ab^	2.88 ± 0.45 ^abc^	209.95 ± 5.45 ^abc^	10.12 ± 0.76 ^abc^

Note: data are shown as mean ± SD, *n* = 12/group. PGE2: prostaglandin E2; NO: nitric oxide. a: means compared with the normal control group, *p* < 0.05; b: means compared with the ethanol group, *p* < 0.05; c: means compared with whey protein group, *p* < 0.05.

## Data Availability

The data presented in this study are available on request from the corresponding author. The data are not publicly available due to privacy.
